# Supplementary material for: The Evolution and Characterization of the RNA Interference Pathways in Lophotrochozoa
Source: Genome Biol Evol. 2024 May 7;16(5):evae098. doi: 10.1093/gbe/evae098 (PMC11114477; doi:10.1093/gbe/evae098)
Supplement: evae098_Supplementary_Data [file evae098_supplementary_data.zip › Supplementary_materials.zip!/selected_miRNAs/NoGe_selected_miRNAs/mirdeep_pdf_files/NMRB01002226.1_Notospermus_geniculatus_strain_Ushimado_scaffold2226,_whole_genome_shotgun_sequence_36874.pdf]

Provisional ID	:	NMRB01002226.1_Notospermus_geniculatus_strain_Ushimado_scaffold2226,_whole_genome_shotgun_sequence_36874
Score total	:	2107
Score for star read(s)	:	3.9
Score for read counts	:	2098.6
Score for mfe	:	1.5
Score for randfold	:	0
Score for cons. seed	:	3
Total read count	:	4128
Mature read count	:	4099
Loop read count	:	0
Star read count	:	29

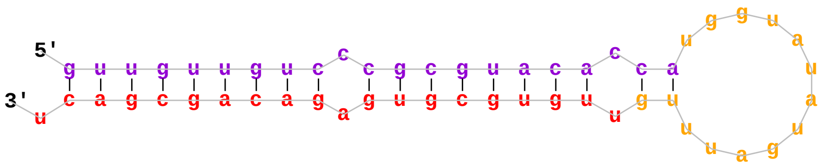

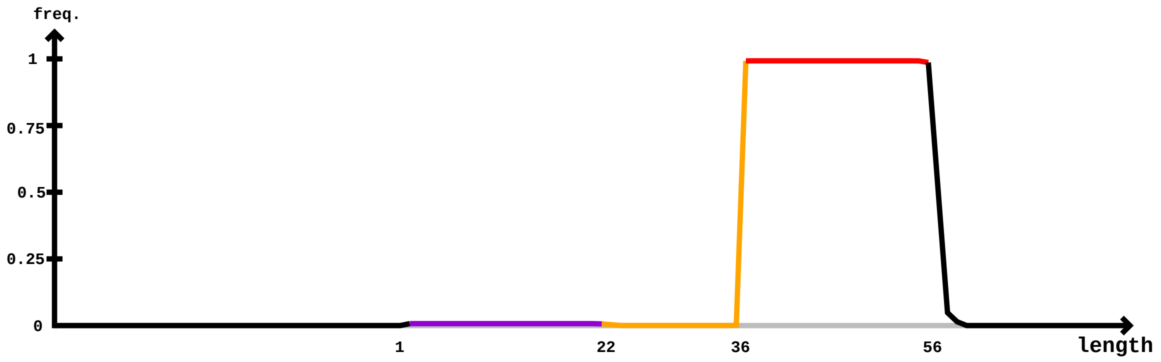

Star **Mature**

```
5'- cggacacggacacauucgagagaggcuucccacuuguuguugucccgcguacaccaugguauaugauuuguugugcgugagacagcgacuaaugugcaagcucauuucau  -3'  obs
    cggacacggacacauucgagagaggcuucccacuuguuguugucccgcguacaccaugguauaugauuuguugugcgugagacagcgacuaaugugcaagcucauuucau       exp
    ((....)).........((((..(((((..(((..(((((((((.((((((((.((............)).)))))))).)))))))))....))).)))))..))))..  reads  mm  sample
    ...................................guuguugucccgcguacacc.......................................................  4      0   Fm1
    ...................................guuguugucccgcguacaccG......................................................  1      1   Fm1
    ...................................guuguugucccgcguacacca......................................................  19     0   Fm1
    ......................................................................uugugcgugagacagcgac.....................  8      0   Fm1
    ......................................................................uugugcgugagacagcUacu....................  1      1   Fm1
    ......................................................................uuCugcgugagacagcgacu....................  1      1   Fm1
    ......................................................................uugugcgugagacagcgacA....................  1      1   Fm1
    ......................................................................uugugcgugagacagcgGcu....................  2      1   Fm1
    ......................................................................uugugcgugagacagcgacG....................  5      1   Fm1
    ......................................................................Nugugcgugagacagcgacu....................  1      1   Fm1
    ......................................................................uugugcgugagacagcgacu....................  240    0   Fm1
    ......................................................................uugugcgugagacagcCacu....................  2      1   Fm1
    ......................................................................uugugcgugaAacagcgacu....................  1      1   Fm1
    ......................................................................uugugcgugagacagcgCcu....................  1      1   Fm1
    ......................................................................uugugcgugagacagcgUcu....................  2      1   Fm1
    ......................................................................uugugcgugagacagcgacuU...................  52     1   Fm1
    ......................................................................uugugcgugagacagcgCcua...................  2      1   Fm1
    ......................................................................uugugcCugagacagcgacua...................  1      1   Fm1
    ......................................................................uugugcgugagacagcgacuC...................  5      1   Fm1
    ......................................................................uugugcgugagacagcUacua...................  1      1   Fm1
    ......................................................................uAgugcgugagacagcgacua...................  1      1   Fm1
    ......................................................................uugugcgugaAacagcgacua...................  1      1   Fm1
    ......................................................................uugugcgugagacagcgacuG...................  3      1   Fm1
    ......................................................................uugugcgugagacagcCacua...................  1      1   Fm1
    ......................................................................uugugcgugagacagcgacua...................  251    0   Fm1
    ......................................................................uugugcgugagacagcAacua...................  1      1   Fm1
    ......................................................................uugCgcgugagacagcgacua...................  1      1   Fm1
    ......................................................................uugugcgugagacagcgUcua...................  1      1   Fm1
    ......................................................................uugugcgugagacagcgacuaa..................  3      0   Fm1
    ......................................................................uugugcgugagacagcgacuGa..................  2      1   Fm1
    ......................................................................uugugcgugagacagcgacuaU..................  24     1   Fm1
    ......................................................................uugugcgugagacagcgacuaUu.................  15     1   Fm1
    ......................................................................uugugcgugagacagcgacuaau.................  3      0   Fm1
```

Star **Mature**

```
cggacacggacacauucgagagaggcuucccacuuguuguugucccgcguacaccaugguauaugauuuguugugcgugagacagcgacuaaugugcaagcucauuucau

......................................................................uugugcgugagacagcgacG....................  1    1  Fm2
......................................................................uugugcgugagacagcgacu....................  37   0  Fm2
......................................................................uugugcgugagacagcUacu....................  1    1  Fm2
......................................................................uugugcgugaUacagcgacu....................  1    1  Fm2
......................................................................uugugcgugagacagcgacuU...................  3    1  Fm2
......................................................................uugugcgugagacagcgCcua...................  2    1  Fm2
......................................................................uugugcgugagacagcgacua...................  113  0  Fm2
......................................................................uugugcgugagacagcgGcua...................  2    1  Fm2
......................................................................uugugcgugaUacagcgacua...................  1    1  Fm2
......................................................................uugugcgugagacagcgacuC...................  2    1  Fm2
......................................................................uugugcgugagacagcgacuaU..................  3    1  Fm2
......................................................................uugugcgugagacagcgacuaUu.................  4    1  Fm2

...................................guuguugucccgcguacacca......................................................  2    0  Mm2
...................................guuguugucccgcguacaccaugguauaugauuug........................................  1    0  Mm2
......................................................................uugugcgugagacagcgac.....................  2    0  Mm2
......................................................................uugugcgugagacagcgCc.....................  1    1  Mm2
......................................................................uugugcgugagacagcgacu....................  102  0  Mm2
......................................................................uugugcgugagacagcgGcu....................  1    1  Mm2
......................................................................uugugcAugagacagcgacu....................  1    1  Mm2
......................................................................uugugcgugagacagcgaUu....................  1    1  Mm2
......................................................................uugugcgugaAacagcgacua...................  1    1  Mm2
......................................................................uugugcgugagacagcgacuU...................  15   1  Mm2
......................................................................uugugcgugagacagcUacua...................  1    1  Mm2
......................................................................uugugcgugagUcagcgacua...................  1    1  Mm2
......................................................................uugugcgugagacagcgacuC...................  2    1  Mm2
......................................................................uugugcgugagacagcgacuG...................  1    1  Mm2
......................................................................uugugcgugagacagcgacua...................  86   0  Mm2
......................................................................uugugcgugagacagcgacuaU..................  8    1  Mm2
......................................................................uugugcgugagacagcgacuaa..................  1    0  Mm2
......................................................................uugugcgugagacagcgacuaG..................  2    1  Mm2
......................................................................uugugcgugagacagcgacuUa..................  1    1  Mm2
......................................................................uugugcgugagacagcgacuaUu.................  2    1  Mm2

......................................................................uugugcgugaUacagcgacu....................  1    1  Mm1
......................................................................Nugugcgugagacagcgacu....................  1    1  Mm1
......................................................................uugugcgugaCacagcgacu....................  1    1  Mm1
......................................................................uugugcgugagacagcgUcu....................  2    1  Mm1
......................................................................uugugcgugagacagcgacu....................  186  0  Mm1
......................................................................uugugcgugagacagcgCcu....................  1    1  Mm1
......................................................................uugugcgugagacagcUacu....................  1    1  Mm1
......................................................................uugugcgugagacagcCacu....................  1    1  Mm1
......................................................................Cugugcgugagacagcgacu....................  1    1  Mm1
......................................................................uugugcgugagacagcgUcua...................  1    1  Mm1
......................................................................uugugcgugagacagcUacua...................  1    1  Mm1
......................................................................uugugcgugagacagcgacuU...................  9    1  Mm1
......................................................................uugugcgugagacagcgacua...................  153  0  Mm1
......................................................................uugugcgugagacagcgacuaU..................  14   1  Mm1
......................................................................uugugcgugagacagcgacuaUu.................  2    1  Mm1
......................................................................uugugcgugagacagcgacuaCu.................  4    1  Mm1

...................................guuguugucccgcguacacca......................................................  2    0  Fm3
......................................................................uugugcgugagacagcga......................  1    0  Fm3
......................................................................uugCgcgugagacagcgac.....................  1    1  Fm3
......................................................................uugugcgugagacagcgac.....................  9    0  Fm3
......................................................................uugugcgugagacagcUacu....................  3    1  Fm3
......................................................................uugugcgugagacagcgCcu....................  6    1  Fm3
......................................................................uugugcgugagacagcgGcu....................  3    1  Fm3
......................................................................uugugcgugagaUagcgacu....................  1    1  Fm3
......................................................................uugugcgugaCacagcgacu....................  1    1  Fm3
......................................................................uugugcgugagacagcCacu....................  5    1  Fm3
......................................................................uugugcgugagacGgcgacu....................  1    1  Fm3
......................................................................uugugcgugaUacagcgacu....................  1    1  Fm3
......................................................................uuguAcgugagacagcgacu....................  1    1  Fm3
......................................................................uugugcgugagacagcAacu....................  2    1  Fm3
......................................................................uugugcgugagacaUcgacu....................  1    1  Fm3
......................................................................uugugUgugagacagcgacu....................  2    1  Fm3
......................................................................uugugcAugagacagcgacu....................  1    1  Fm3
......................................................................uugugcgugaAacagcgacu....................  1    1  Fm3
```

```
                                   Star                             Mature
cggacacggacacauucgagagaggcuucccacuuguuguugucccgcguacaccaugguauaugauuuguugugcgugagacagcgacuaaugugcaagcucauuucau
......................................................................uugugcgugagacagcgacu....................    1313    0    Fm3
......................................................................uugugcgugagacagcgUcu....................    6       1    Fm3
......................................................................Nugugcgugagacagcgacu....................    1       1    Fm3
......................................................................uuUugcgugagacagcgacua...................    1       1    Fm3
......................................................................uugugcgugagacagcgacuC...................    2       1    Fm3
......................................................................uugugcgugagacagcgacua...................    830     0    Fm3
......................................................................uugugAgugagacagcgacua...................    1       1    Fm3
......................................................................uugugcgugagacagcgacuU...................    74      1    Fm3
......................................................................uugugcgugagacagcgCcua...................    4       1    Fm3
......................................................................uugugcgugagacagcCacua...................    1       1    Fm3
......................................................................Gugugcgugagacagcgacua...................    1       1    Fm3
......................................................................uugugcgugaAacagcgacua...................    1       1    Fm3
......................................................................uugugcgugagacagcgGcua...................    3       1    Fm3
......................................................................uugugcgugagacagcUacua...................    1       1    Fm3
......................................................................uugugcgugagacagcgUcua...................    3       1    Fm3
......................................................................uugugcguAagacagcgacua...................    2       1    Fm3
......................................................................uugugUgugagacagcgacua...................    1       1    Fm3
......................................................................uugugcgugagacagcgaGua...................    1       1    Fm3
......................................................................uugugcgugagacUgcgacua...................    1       1    Fm3
......................................................................uugugcCugagacagcgacua...................    1       1    Fm3
......................................................................uugugcgugagacagcgacuaC..................    5       1    Fm3
......................................................................uugugcgugagacagcgacuUa..................    5       1    Fm3
......................................................................uugugcgugagacagcgacuaU..................    51      1    Fm3
......................................................................uugugcgugagacagcgacuaa..................    10      0    Fm3
......................................................................uugugcgugagacagcgacuaaG.................    2       1    Fm3
......................................................................uugugcgugagacagcgacuaaA.................    4       1    Fm3
......................................................................uugugcgugagacagcgacuaau.................    4       0    Fm3
......................................................................uugugcgugagacagcgacuaCu.................    1       1    Fm3
......................................................................uugugcgugagacagcgacuaUu.................    14      1    Fm3
.......................................................................ugugcgugagacagcgacu....................    1       0    Fm3

...................................guuguugucccgcguacacca......................................................    1       0    Mm3
.....................................................................guugugcgugagacagcgacu....................    1       0    Mm3
......................................................................uugugcgugagacagcgac.....................    4       0    Mm3
......................................................................uugugcgugagacagcgacu....................    133     0    Mm3
......................................................................uuCugcgugagacagcgacu....................    1       1    Mm3
......................................................................uugugcgugagacagcgGcu....................    1       1    Mm3
......................................................................Augugcgugagacagcgacu....................    1       1    Mm3
......................................................................uugugcgugagacagcgacG....................    1       1    Mm3
......................................................................uugugcgugagacagcgacua...................    125     0    Mm3
......................................................................uugugcgugagacagcgUcua...................    1       1    Mm3
......................................................................uugugcgugagacagcgGcua...................    1       1    Mm3
......................................................................uugugcgugagacagcgacuU...................    17      1    Mm3
......................................................................uugugcgugagacagcgacuC...................    1       1    Mm3
......................................................................uugugcgugagacaCcgacua...................    1       1    Mm3
......................................................................uugugcgugagacagcgacuaU..................    10      1    Mm3
......................................................................uugugcgugagacagcgacuCa..................    1       1    Mm3
......................................................................uugugcgugagacagUgacuaau.................    1       1    Mm3
......................................................................uugugcgugagacagcgacuaUu.................    2       1    Mm3
......................................................................uugugcgugagacagcgacuaaA.................    1       1    Mm3
```
